# Coexisting ovarian and tubal pregnancies on opposite sides after intrauterine insemination: a case report

**DOI:** 10.1186/s12884-018-1801-6

**Published:** 2018-06-25

**Authors:** Jeong Min Eom, Joong Sub Choi, Jaeman Bae, Won Moo Lee, Eunhyun Lee, Jongwon Lee, Ji Hyun Keum

**Affiliations:** Division of Gynecologic Oncology and Gynecologic Minimally Invasive Surgery, Full time faculty of the Department of Obstetrics and Gynecology, Hanyang University College of Medicine, 222-1 Wangsimni-ro Seongdong-gu, Seoul, 04763 Republic of Korea

**Keywords:** Ovarian pregnancy, Ectopic pregnancy, Laparoscopy, Surgery

## Abstract

**Background:**

Ovarian pregnancy is very rare, and contralateral tubal pregnancy coexisting with ovarian pregnancy must be even rarer.

**Case presentation:**

A 33-year-old Korean nulliparous woman was referred to our hospital because she suffered lower abdominal pain and had missed her periods after controlled ovarian hyperstimulation and intrauterine insemination. We could not identify any normal gestational sac in the endometrium, or specific ectopic pregnancies, on an initial ultrasound scan. However, there was a large hematoma in the cul-de-sac and free fluid in the right paracolic gutter. We decided to perform emergent laparoscopic surgery. We found contralateral tubal and ovarian ectopic pregnancies.

**Conclusion:**

To the best of our knowledge, this is the first report of a case in which a patient underwent laparoscopic right salpingectomy and left ovarian ectopic mass excision due to contralateral tubal and ovarian ectopic pregnancies after assisted reproductive technology.

**Electronic supplementary material:**

The online version of this article (10.1186/s12884-018-1801-6) contains supplementary material, which is available to authorized users.

## Background

The recent increase in use of assisted reproductive technology (ART) has contributed to the occurrence of various types of ectopic pregnancy [1, 2]. Most of these are restricted to the fallopian tubes, ovary, cervix, and abdominal cavity, and intrauterine and extrauterine pregnancies can occur simultaneously as in heterotopic pregnancy [1, 3, 4]. Also, very infrequently, ectopic pregnancies can occur simultaneously at different extrauterine sites [5]. Spontaneous double or multiple ovulations rarely occurs in humans. However, as multiple ovulation is possible after ART, two or more simultaneous ectopic pregnancies can occur [5, 6]. Most of the simultaneous ectopic pregnancies reported so far occurred in the fallopian tubes; in other words, they were bilateral tubal pregnancies [5].

The most common cause of hemoperitoneum in early pregnancy is ectopic pregnancy, which can be life-threatening and requires early accurate diagnosis. In a rare type of ectopic pregnancy, hemoperitoneum caused by rupture makes it difficult to diagnose the ectopic pregnancy before surgery. As a result, ectopic pregnancy complicated by hemoperitoneum is frequently discovered during surgery [7, 8].

We performed emergent laparoscopic management in a patient who had significant hemoperitoneum after controlled ovarian hyperstimulation and intrauterine insemination, and discovered an ovarian pregnancy on the left side and tubal pregnancy in the right side. We report the first case of this kind.

## Case presentation

A 33-year-old nulliparous woman was referred to our institution from a private infertility clinic complaining of lower abdominal pain. She reported a history of 5 weeks and 4 days of amenorrhea and had undergone intrauterine insemination (IUI) 27 days previously. Ovarian hyperstimulation for IUI was started with Clomiphene citrate 100 mg daily during the 3rd–7th days of the menstrual cycle, followed by 75 IU hMG (IVF-M HP, LG life science, Seoul, Korea) daily on the 7th–9th days of the menstrual cycle. Transvaginal ultrasound had revealed four dominant follicles in the left ovary after ovarian stimulation.

**Fig. 1:**
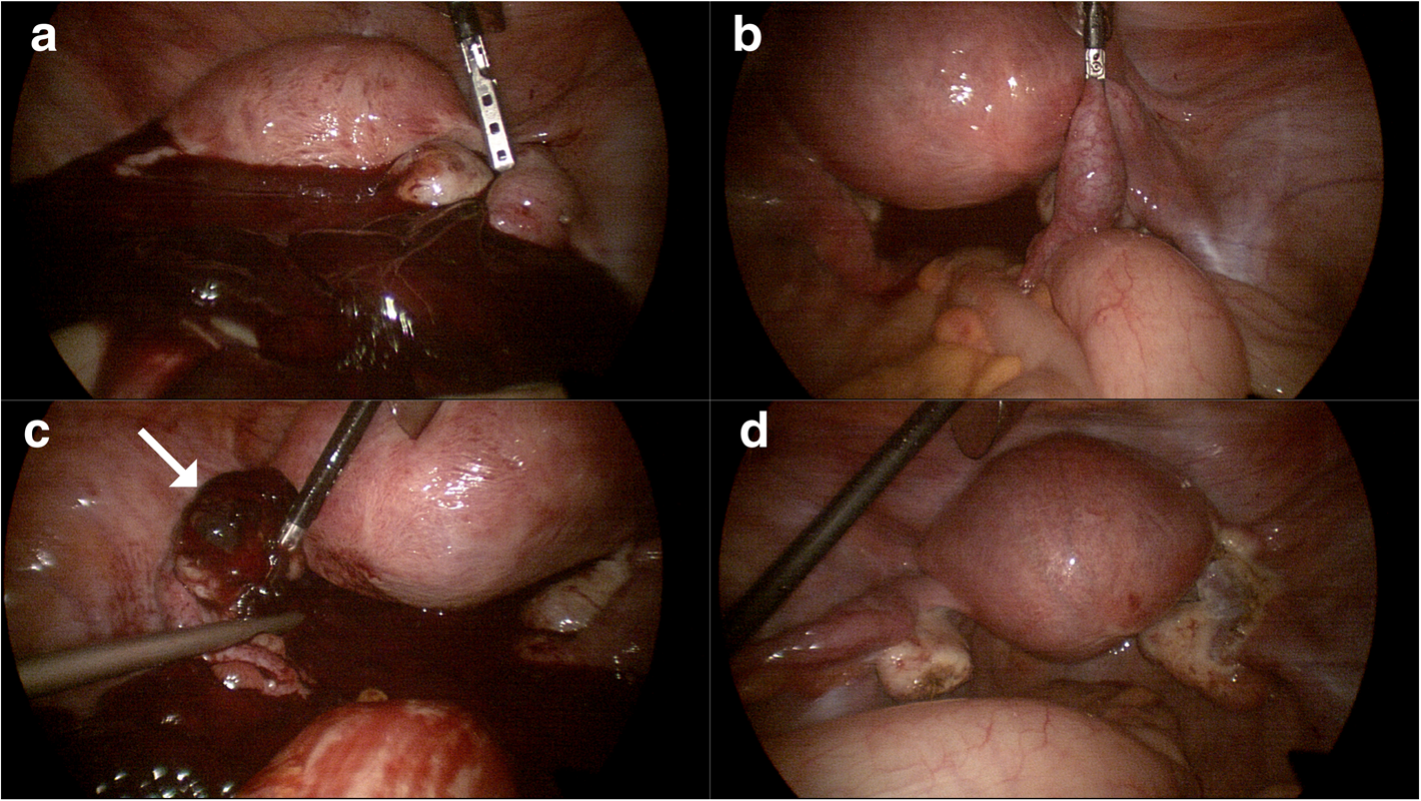
**a** Laparoscopic operative findings reveal a hematoma and hemoperitoneum in the pelvic cavity. **b** The right tubal pregnancy. **c** The left ruptured ovarian pregnancy (white arrow). **d** Appearance of the pelvic cavity after right salpingectomy and removal of the left ovarian pregnancy

**Fig. 2:**
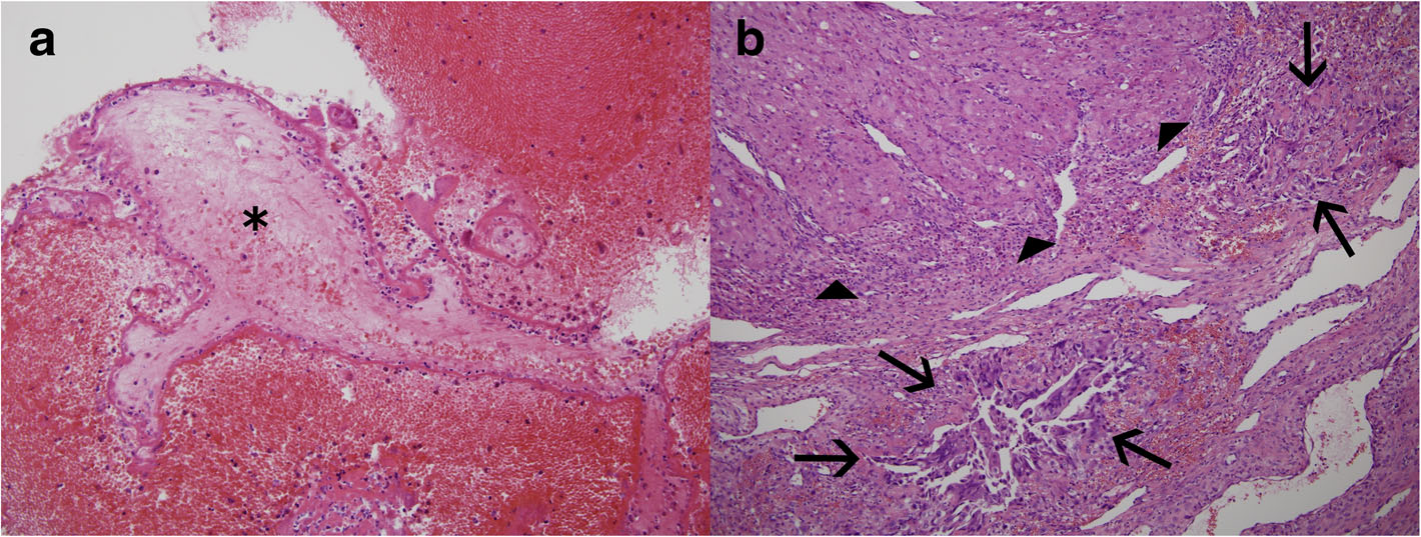
A representative microphotograph of the surgical specimen. **a** The fallopian tube contains fresh blood clots with necrotic chorionic villi (asterisks). **b** In the ovary can be seen the corpus luteum (arrow heads) with infiltrating intermediate trophoblasts (arrows) in the ovarian parenchyma

On physical examination, she had normal vital signs and diffuse lower abdominal tenderness. The serum beta-chorionic gonadotropin level was 3154 mUI/mL. Transvaginal ultrasound performed in the gynecology department revealed a large hyperechoic mass, a suspected hematoma, in the cul-de-sac. It also revealed a normal-sized uterus without an intrauterine gestational sac, and endometrial thickening of 20 mm. Both right and left adnexa were normal on the ultrasound. The initial complete blood count was as follows: hematocrit 35.9%, hemoglobin 11.9 g/dL, white blood cells 9.3 × 10^9^/L and platelets 252 × 10^9^/L. The provisional diagnosis was ruptured ectopic pregnancy with hemoperitoneum, and emergency laparoscopy was performed. Intraoperatively, a dark blood clot of about 800 ml was seen along with a small amount of fresh blood (Fig. 1a). An approximately 2 × 2 × 1.5 cm unruptured ectopic pregnancy was found in the right fallopian tube (Fig. 1b), while the left fallopian tube appeared to be normal. While examining the ovaries to locate the cause of the bleeding, we observed minimal bleeding from the proximal pole of the left ovary, where there was a 1.0 × 0.5 × 0.5 cm hemorrhagic mass with surrounding blood clot suggestive of a ruptured ectopic pregnancy (Fig. 1c). We resected the mass from the left ovary and performed a right salpingectomy using monopolar electrocautery and 5 mm multi-functional bipolar electrocoagulation forceps (LiNA PowerBlade; LiNA Medical, Glostrup, Hovedstaden DK-2600, Denmark) (Fig. 1d). After intraperitoneal irrigation with 3 L of saline, we ascertained that there were no abnormal findings. We therefore placed drains into the cul-de sac and finished the operation (Additional file 1). Histopathological analysis demonstrated a left ovarian pregnancy with presence of chorionic villi within the ovarian tissue, along with a right tubal pregnancy (Fig. 2).

## Discussion

The occurrence of the concomitant ovarian and tubal pregnancies in this case can be explained in the following way. After ovarian hyperstimulation there were four dominant follicles in the left ovary only, and an ovum released from the left ovary was fertilized in the left fallopian tube; however the conceptus then refluxed to the left ovary and was implanted in it, leading to the left ovarian pregnancy [9]. The right tubal pregnancy, on the other hand, was caused by contralateral pick-up by the right fallopian tube of an ovum from the left ovary. Contralateral ectopic pregnancy due to ovum transmigration as in this case is reported to occur in 15–60% of tubal pregnancies [10]. Ovarian pregnancy is very rare, representing only 0.5–3% of all ectopic pregnancies. Hence, contralateral tubal pregnancy coexisting with ovarian pregnancy must be even rarer [11].

Ovarian pregnancy is not easy to diagnose [12–15]. In our case, it occurred simultaneously with tubal pregnancy, and was especially difficult to diagnose because it was accompanied by hemoperitoneum. When the cause of bleeding is hard to discover and if there is hematoma in the abdominal cavity, it is necessary to perform laparoscopic surgery as soon as possible even if vital signs are stable and there are no symptoms [12]. Because the patient had a hematoma with abdominal pain, we performed emergent laparoscopic surgery to obtain an accurate diagnosis and alleviate the symptoms, and a right tubal pregnancy was encountered during the surgery. A small mass in the left ovary, which was surrounded by a hematoma and showed minimal bleeding, was the reason for the hemoperitoneum, and was also not detected by ultrasound image before the surgery. In the histopathologic report, we considered the possibility that the mass in the left ovary was a ruptured corpus luteal cyst and the bleeding to be due to its rupture. Because an ovarian pregnancy is similar to any other ovarian cyst, preoperative diagnosis is not easy and is even more difficult to diagnose if its rupture is accompanied by simultaneous hemorrhage and hematoma [9]. Because the left ovarian tissue removed during surgery had been damaged by the rupture its original shape was not clear, and it was hard to diagnose the ectopic pregnancy before the final histopathologic report, which identified chorionic villi attached to the ovarian tissue and pointed to a diagnosis of ovarian pregnancy (Fig. 2b). In cases where the exact location of an ectopic pregnancy is not identified on ultrasound scan preoperatively, in the presence of hemoperitoneum, laparoscopy is the most useful method because the whole abdomen can be examined and simultaneous diagnosis and treatment are possible [16].

## Conclusion

We report for the first time the simultaneous occurrence of an ovarian and a tubal pregnancy on different sides of the uterus after controlled ovarian hyperstimulation and intrauterine insemination. Because there had been no previous report of this situation, it was hard to account for it before surgery was undertaken, and it needed to be distinguished from other causes that accompany abdominal pain and hemoperitoneum. Laparoscopic surgery was very effective in providing an exact diagnosis and permitting prompt surgical intervention in this rare case.

## Additional file

Additional file 1: Timeline of treatment. This figure shows timeline of treatment for this case. (PDF 123 kb) (PDF 122 kb)

## Abbreviations

ART: Assisted reproductive technology (ART)
IUI: Intrauterine insemination
