# Supplementary material for: Coexisting ovarian and tubal pregnancies on opposite sides after intrauterine insemination: a case report
Source: BMC Pregnancy Childbirth. 2018 Jun 25;18:259. doi: 10.1186/s12884-018-1801-6 (PMC6019221; doi:10.1186/s12884-018-1801-6)
Supplement: Supplementary file 1 — Timeline of treatment. This figure shows timeline of treatment for this case. (PDF 123 kb) (PDF 122 kb) [file 12884_2018_1801_MOESM1_ESM.pdf]

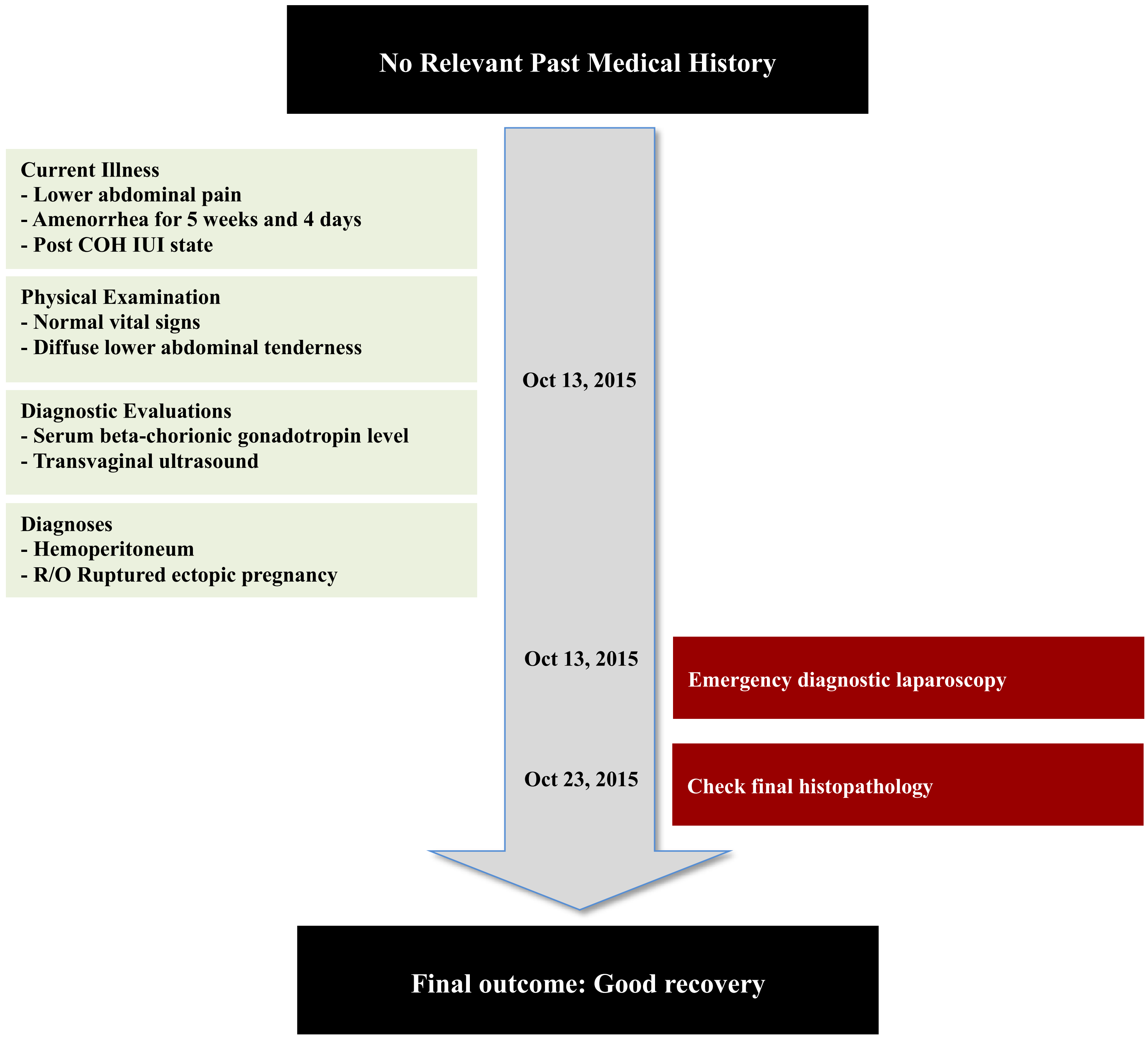
No Relevant Past Medical History
Current Illness
- Lower abdominal pain
- Amenorrhea for 5 weeks and 4 days
- Post COH IUI state
Physical Examination
- Normal vital signs
- Diffuse lower abdominal tenderness
Diagnostic Evaluations
- Serum beta-chorionic gonadotropin level
- Transvaginal ultrasound
Diagnoses
- Hemoperitoneum
- R/O Ruptured ectopic pregnancy
Oct 13, 2015
Oct 13, 2015
Emergency diagnostic laparoscopy
Oct 23, 2015
Check final histopathology
Final outcome: Good recovery
